# Delayed neurological recovery in ischemic stroke patients undergoing endovascular treatment is associated with baseline hyperglycemia: a treatable cause of the stunned brain phenomenon?

**DOI:** 10.1007/s00415-025-13019-x

**Published:** 2025-04-04

**Authors:** Susan Klapproth, Lukas Meyer, Helge Kniep, Matthias Bechstein, Anna Kyselyova, Susanne Gellißen, Christian Heitkamp, Laurens Winkelmeier, Uta Hanning, Gerhard Schön, Marlene Heinze, Karolin Schulte, Jens Fiehler, Gabriel Broocks

**Affiliations:** 1 Department of Diagnostic and Interventional Neuroradiology, University Medical Center Hamburg-Eppendorf, Martinistrasse 52, 20246 Hamburg, Germany; 2 Institute of Medical Biometry and Epidemiology, University Medical Center Hamburg-Eppendorf, Hamburg, Germany; 3 Department of Neurology, University Medical Center Hamburg-Eppendorf, Hamburg, Germany; 4 Department of Neuroradiology, HELIOS Medical Center, Campus of MSH Medical School Hamburg, Schwerin, Germany; 5 Department of Neuroradiology, University Hospital and University of Zurich, Zurich, Switzerland

**Keywords:** Stroke, NWU, Blood glucose levels

## Abstract

**Background and aims:**

In ischemic stroke, there is limited data regarding the impact of baseline hyperglycemia on the treatment effect of recanalization on neurological recovery. This study aimed to directly compare—how short- and long-term serum glucose levels modify the effect of recanalization on functional outcome in patients with ischemic stroke and specifically analyze the occurrence of delayed neurological recovery (“stunned brain phenomenon”).

**Methods:**

Observational retrospective analysis including patients with anterior circulation ischemic stroke and large vessel occlusion undergoing mechanical thrombectomy following multimodal-CT upon admission. The primary endpoint was delayed neurological recovery, defined as a lack of early neurological improvement (ENI) at 24 h despite achieving functional independence at day 90. Binary ENI was defined as 24 h-NIHSS ≤ 8 points. The treatment effect of recanalization defined as mTICI 2b-3 was determined for patients with high versus low serum blood glucose (BG, cut-off: 140 mg/dl). Inverse-probability weighting analysis (IPW) was used to assess the treatment effect of recanalization according to glucose profiles.

**Results:**

A total of 348 patients were included in the analysis. The treatment effect of recanalization in patients with low BG on the NIHSS at 24 h and binary ENI was  – 3.5 (95%CI  – 5.3 to  – 1.8, *p* < 0.001) and 22.4% (95%CI 13.1–31.8, *p* < 0.001). Furthermore, recanalization in patients with low BG was associated with functional independence at day 90 (26.4%, 95%CI 17.1–35.8, *p* < 0.001). For patients with high BG, recanalization was not associated with a lower NIHSS at 24 h ( – 1.4, 95%CI  – 3.7–0.9, *p* = 0.24) although significantly being associated with functional independence at day 90 (+ 14.7%, 95%CI 4.5–24.9, *p* = 0.005).

**Discussion:**

Successful vessel recanalization was associated with better functional outcome at day 90 independent of BG profiles; however, acute hyperglycemia was significantly linked to delayed neurological recovery. Hence, hyperglycemia might be a major cause of the stunned brain phenomenon and might consequently serve as a promising target for adjunctive therapy in the treatment of ischemic stroke patients.

## Introduction

Mechanical thrombectomy [1] is the standard of care for patients with ischemic stroke caused by a large vessel occlusion [2, 3]. Still, a high proportion of patients, even after complete revascularization, do not achieve functional independence. Several factors beyond reperfusion are known to be predictive of functional outcomes, such as age or collateral status [4, 5]. Currently, several variables are investigated that may be used as further treatment targets to improve outcome, for instance, the blood glucose level (BG) that are assessed for every stroke patient upon admission. Recently, the CHARM trial assessed the administration of intravenous glibenclamide, an antidiabetic drug known to be associated with reduced ischemic edema formation in stroke, on functional outcomes, in stroke patients [6]. While it is known that higher BG is associated with worse clinical outcomes, there is limited data on the pathophysiological relationship of BG, ischemic lesion evolution, and outcome, and how BG modifies the effect of endovascular recanalization [7, 8].

As the final functional outcome at day 90 can not always be assessed after patient discharge and the functional status following stroke may also be directly related to secondary complications such as hospital-acquired pneumonia, particularly in elderly or comorbid patients, further endpoints evaluating treatment effects in stroke are required. In this context, early neurological improvement (ENI), assessed 24 h after admission has been proposed as an NIHSS-based approach to screen ischemic stroke patients, and it has been observed that ENI at 24 h is well-correlated with the final functional status at day 90 [9]. Nevertheless, a high proportion of patients may achieve good functional outcomes. Despite a poor functional status at 24 h. This phenomenon has been described as “stunned brain phenomenon”. Several variables have been associated with this phenomenon such as the extent of infarction and involvement of specific brain areas (motor cortex, internal capsule), as well as higher premorbid modified Rankin scale, end-stage renal failure, or absence of bridging intravenous lysis. The identification of variables associated with delayed neurological recovery is of high relevance as a modification of these parameters may directly improve functional outcome and response to endovascular recanalization. Yet, it has not been assessed to which degree short versus long-term (i.e., glycated hemoglobin, HbA1c) BG modifies the effect of recanalization on ENI and final functional outcome at day 90. We hypothesized that elevated BG and HbA1c levels are associated with delayed neurological recovery in ischemic stroke following endovascular recanalization.

## Methods

### Study cohort

#### Patients

We conducted a retrospective analysis of ischemic stroke patients with large vessel occlusion in the territory of the middle cerebral artery. These patients received treatment at a university stroke center from 01/2015 to 01/2022. Ethical approval was obtained before recording anonymized data (Ethikkommission der Ärztekammer Hamburg (WF0413). The study was performed following the Declaration of Helsinki. The corresponding author can provide data supporting the study results upon reasonable request.

The inclusion criteria established a priori were:

1. Acute ischemic stroke with large vessel occlusion of the distal internal carotid artery or middle cerebral artery (MCA) confirmed by multimodality CT on admission (nonenhanced CT (NECT), CT angiography (CTA), and CT perfusion (CTP)); a visually apparent early infarct lesion is identified by ischemic hypoattenuation in the initial non-contrast-enhanced tomography (NECT) upon admission and/or a lesion on perfusion computed tomography (CTP) accompanied by reduced cerebral blood flow in the territory of the MCA; subsequently performed endovascular procedure with documented mTICI score, mTICI ≥ 2b defined as successful recanalization (modified Thrombolysis in Cerebral Infarcts); absence of intracranial hemorrhage on admission NECT; complete documentation of serum glucose levels (BG) on admission and glycated hemoglobin (HbA1c).

Baseline clinical data and demographic information were obtained from medical records, including blood glucose level on admission and HbA1c level as well as antidiabetic medication, including insulin. Functional outcome was taken from the registry using the modified Rankin Scale (mRS) at 90 days.

A history of diabetes mellitus was retrieved from clinical documentation. Elevated BG was defined as ≥ 140 mg/dl according to the current recommendations of the Centers for Disease Control and Prevention (CDC). HbA1c thresholds were defined as < 5.7% (normal), 5.7–6.4% (elevated), and ≥ 6.5% (strongly elevated) [14–16]. The association of short versus long-term BG profiles and outcomes after recanalization were compared. The primary endpoint was delayed neurological recovery, defined as a lack of ENI despite achieving functional independence at day 90. ENI was defined two-fold 1) binarized according to Meyer et al. as NIHSS improvement ≥ 8 points from baseline or reaching ≤ 1[10], and using absolute NIHSS changes from baseline to follow-up (24 h after admission). Intracranial arterial collaterals were assessed using the Maas System [11] with favorable collateral scores defined as > 2.

### Statistical analysis

We used standard descriptive statistics to present all data. Normal distribution was assessed through Shapiro–Wilk tests. Continuous variables are expressed as means with confidence intervals (CI) or standard deviation (SD), or as medians and interquartile ranges (IQR). For comparing groups of patients with ENI versus those without, Student *t*-tests (for normal distribution) with confidence intervals (CI) or Mann–Whitney *U* tests (for non-normal distribution) with interquartile range (IQR) were utilized (see Table 1). The categorical variables were compared using χ2 tests.

**Table 1:** Patient characteristics

Baseline characteristics	Functional independence	mRS 3–6	*P* value
Subjects, *n* (%)	191 (45)	294 (69)	
*Baseline variables*			
Age in years, median (IQR)	68 (59–79)	77 (68–85)	< 0.001
Female sex, *n* (%)	(54)	(43)	0.03
Admission NIHSS, median (IQR)	9 (7–12)	16 (13–20)	< 0.001
ASPECTS, median (IQR)	8(7–9)	7(6–9)	< 0.001
Blood glucose in mg/dl, Median (IQR)	120 (101–131)	138 (106–154)	< 0.001
HbA1c in %, median (IQR)	5.7 (5.4–6.1)	5.8 (5.4–6.4)	0.15
*Treatment and endpoints*			
IVT administration, *n* (%)	87 (60)	135 (51)	0.002
mTICI 2b/3, *n* (%)	107 (87)	154 (68)	< 0.001
mRS, median (IQR)	1(0–2)	6(3–6)	< 0.001

To evaluate the impact of recanalization on ENI and functional independence, inverse probability weighting (IPW) was employed using a doubly robust method. This involved a combined modeling of the treatment and logit outcome (ENI and functional independence) with robust standard error type and a 1e-5 tolerance for overlap assumptions. Covariates included ASPECTS, NIHSS score, age, time from onset to imaging, and recanalization status [1]. For visual illustration, multivariable logistic regression models were used in analogy to the IPW models with functional outcome (binary ENI, functional independence as dependent variables) applying the same independent variables. Multivariable linear regression analysis was used to assess the association of recanalization and absolute NIHSS changes from baseline to follow-up adjusted for age, NIHSS, and ASPECTS. A statistically significant difference was considered at a p-value less than 0.05. The analyses were conducted using Stata/MP 17.0 (Stata Corp, TX, USA).

## Results

485 patients were included in the study, of which 172 were men (49%). The mean age was 74 (SD: 15) and the median NIHSS was 15 (IQR: 9–20). The median time from onset to imaging was 2.7 h (IQR: 1.3–5.0), and the median ASPECTS was 8 (IQR: 6–9). The median arterial collateral score was 3 (IQR: 2–3). The median BG was 122 mg/dl (IQR: 105–148), and the median HbA1c was 5.7 (IQR: 5.4–6.2). 245 patients (70%) were successfully recanalized (mTICI 2b-3).

Comparing patients with functional independence to patients with mRS 3–6 at day 90, we observed a significant difference in the median BG, which was 120 mg/dl (IQR: 101–131) in patients with better outcome and 138 mg/dl (IQR: 106–154) in patients with worse outcome (*p* < 0.001). In contrast, the median HbA1c was not statistically different in both group (5.7 versus 5.8) (Table 1).

The treatment effect of recanalization defined as mTICI 2b-3 on short- and long-term functional outcomes was assessed using IPW analysis (dependent variable: functional independence at day 90 defined as mRS 0–2, and binary ENI at 24 h). Recanalization was significantly associated with functional independence (16.5%, 95%CI 9.7–23.3, *p* < 0.001) and binary ENI (11.1%, 95%CI 4.4–17.9, *p* = 0.001).

### Short-term impact of blood glucose levels on the treatment effect of recanalization

Subsequently, the treatment effect of recanalization on short-term functional outcome for patients with high versus low serum BG was analyzed (cut-off: 140 mg/dl). In patients with low BG, the average treatment effect (ATE) of recanalization on the NIHSS at 24 h was  – 3.5 (95%CI  – 5.3 to  – 1.8, *p* < 0.001). Similarly, binary ENI occurred more often in patients with successful recanalization and low BG (+ 22.4%, 95%CI 13.1–31.8, *p* < 0.001). After 24 h, a significant reduction of infarct volume was observed for low BG patients after successful MT ( – 30.8 ml, 95%CI  – 58.6 to  – 21.0, *p* < 0.001).

For patients with high BG, recanalization was not associated with better outcome at 24 h (absolute NIHSS difference:  – 1.4, 95%CI  – 3.7 to 0.9, *p* = 0.24; binary ENI: + 6.7%, 95%CI  – 4.7 to 18.2, *p* = 0.25). There was a trend towards lower infarct volumes for recanalized patients with high BG ( – 24.4 ml, 95%CI  – 49.1 to 0.25, *p* = 0.05, see Table 2). When differentiating patients according to the median HbA1c, a significant treatment effect of recanalization on short-term outcomes was observed for all subgroups, both in the high and low HbA1c group (cut-off: 5.7%).

**Table 2:** Impact of glucose parameters on imaging and clinical endpoints

ATE Recanalization, Mean (95%CI)	High serum BG (> 140 mg/dl)	Low serum BG (< 140 mg/dl)	High HbA1c (> 5.7%)	Low HbA1c (< 5.8%)
NIHSS at 24 h	– 1.4 (– 3.7 to 0.92) *P* = 0.24	– 3.51 (– 5.3 to – 1.8) *p* < 0.001	– 1.9 (– 3.5 to – 0.22) *P* = 0.03	– 4.47 (– 7.1 to – 1.8) *P* = 0.001
Binary ENI at 24 h	+ 6.7% (– 4.7 to 18.2) *P* = 0.25	+ 22.4% (13.1–31.8) < 0.001	+ 10.6% (22.1–18.9) *p* = 0.01	+ 31.8% (17.6–46.0) *P* < 0.001
mRS 90	– 0.62 (– 1.1 to – 0.1) 0.01	– 1.21 (– 1.63 to – 0.81) *p* < 0.001	– 0.79 (– 1.15 to – 0.43) *P* < 0.001	– 1.45 (– 2.15 to – 0.76) *P* < 0.001
Functional independence (mRS 0–2)	+ 14.7% (4.5–24.9) *p* = 0.005	+ 26.4% (17.1–35.8) *p* < 0.001	+ 19.6% (11.7–27.4) *p* < 0.001	+ 23.4% (7.5–39.4) *p* = 0.004
*Imaging endpoints*				
Total infarct volume in ml	– 24.4 ml (– 49.1 to 0.25) *p* = 0.05	– 30.8 ml (– 58.6 to – 21.0) *p* < 0.001	– 34.4 ml (– 52.2 to – 16.5) *p* < 0.001	– 24.9 ml (– 49.5 to – 0.41) *p* = 0.046
PSV	+ 38.9 ml (12.9–64.9) *p* = 0.003	+ 58.8 ml (36.0–81.6) *p* < 0.001	+ 50.9 ml (30.4–71.5) *p* < 0.001	+ 50.5 ml (18.9–82.0) *P* = 0.002

In multivariable linear regression analysis, recanalization was independently associated with absolute NIHSS improvement (ß:  – 1.6, *p* = 0.007) adjusted for ASPECTS, NIHSS, age, and baseline serum BG. In patients with a low serum BG, recanalization was also independently associated with NIHSS improvement (ß:  – 2.7, *p* < 0.001), while recanalization was not associated with NIHSS improvement (ß:  – 0.3, *p* = 0.8) in patients with a high serum BG. The interaction between recanalization and serum BG was significant (ß: 0.03, *p* = 0.01).

### Long-term impact of blood glucose levels on the treatment effect of recanalization

A significant treatment effect of recanalization on functional outcome at day 90 was observed for patients with high BG (ATE on functional independence + 14.7%, 95%CI 4.5–24.9, *p* = 0.005). Similarly, recanalization was associated with higher rates of functional independence for low BG patients following IPW (+ 26.4%, 95%CI 17.1–35.8, *p* < 0.001). When differentiating patients according to the median HbA1c, a significant treatment effect of recanalization on long-term outcome was observed for all subgroups, both in the high and low HbA1c group (cut-off: 5.7%; + 19.6% for patients with high HbA1c, *p* < 0.001, and + 23.4%, *p* = 0.004 for patients with low HbA1c, respectively).

## Discussion

**Fig. 1:**
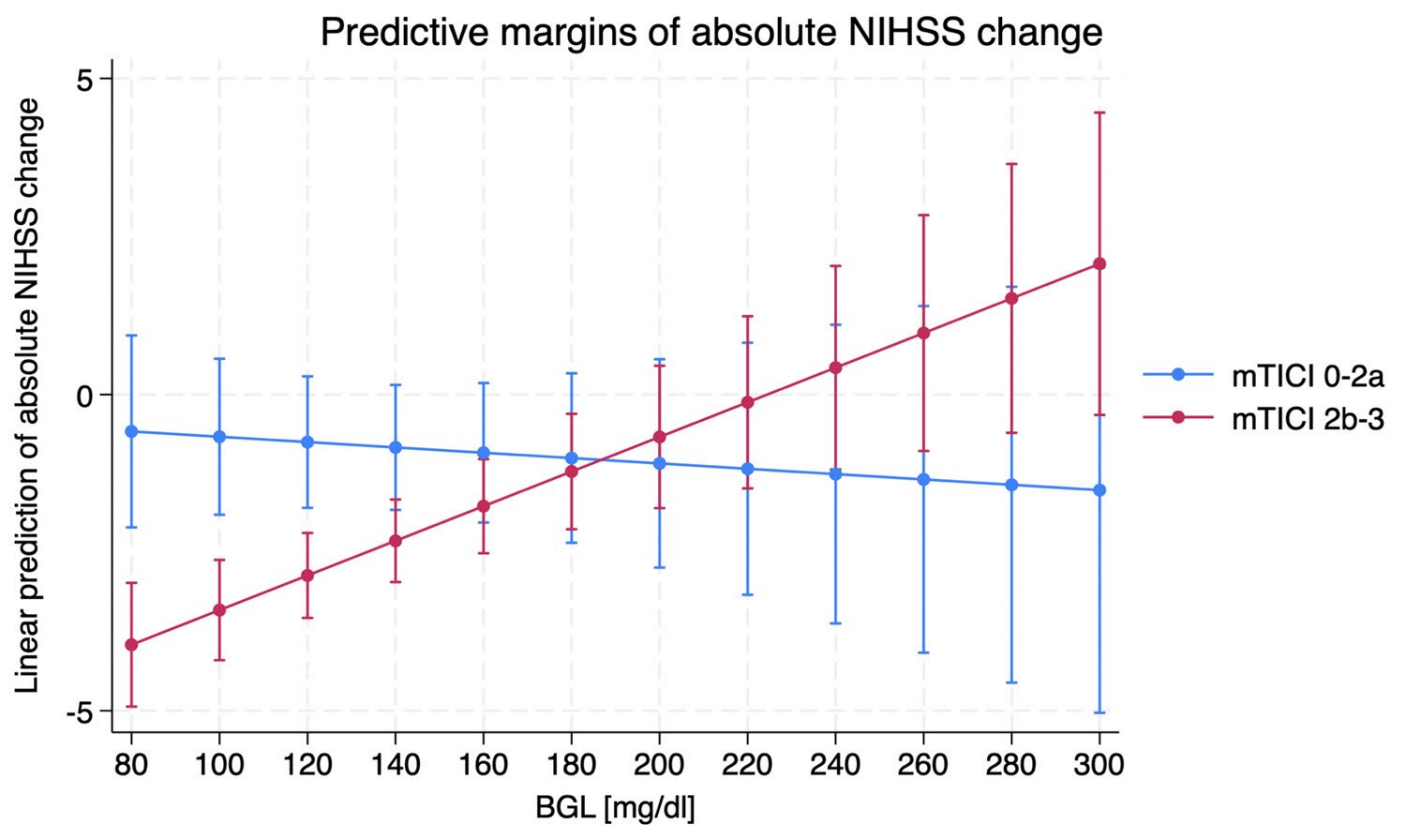
Impact of blood glucose levels on NIHSS changes depending on treatment effect of recanalization defined as mTICI. Multivariate linear regression analysis which displays the impact of blood glucose (x-axis) on NIHSS changes (y-axis) depending on the treatment effect of recanalization (good Tici score defined as Tici 2b-3 and poor Tici score defined as Tici 0-2a)

**Fig. 2:**
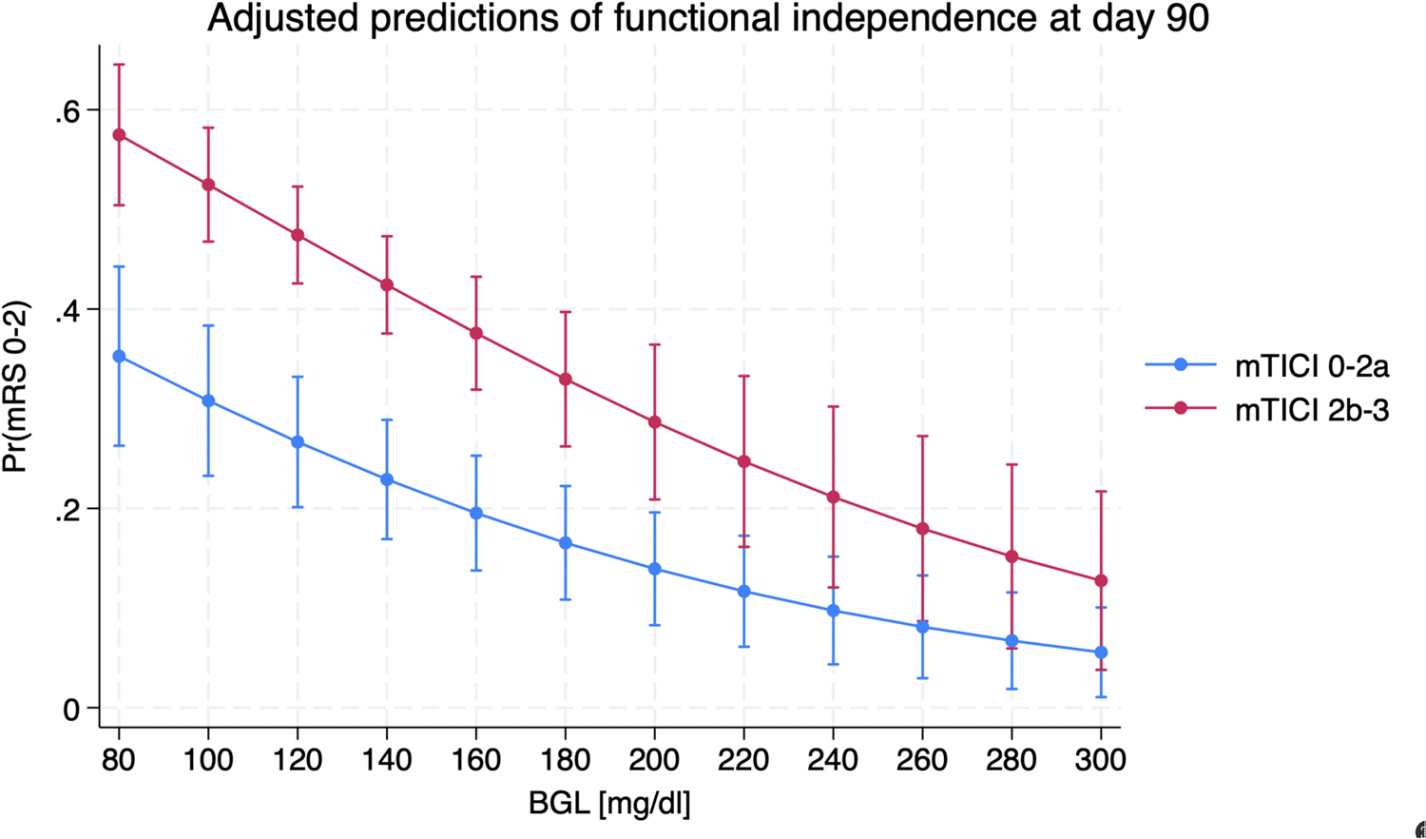
Impact of blood glucose levels on modified Rankin scale depending on the treatment effect of recanalization defined as mTICI. Multivariate linear regression analysis which displays the impact of blood glucose (x-axis) on modified Rankin scale (mRS, y-axis) depending on the treatment effect of recanalization (good Tici score defined as Tici 2b-3 and poor Tici score defined as Tici 0-2a)

This study aimed to investigate how glucose levels in ischemic stroke modify the effect of recanalization on functional outcomes and specifically analyze the occurrence of the stunned brain phenomenon. The main finding of the study was that recanalization was significantly associated with better short- and long-term functional outcomes in patients with low serum BG on admission; however, there was no significant treatment effect on absolute and binarized ENI in patients with high serum BG. As shown in Fig. 1, the effect of thrombectomy decreases with increasing serum BG in relation to the NIHSS changes, with confidence intervals overlapping above a serum BG of 140, while the effect on mRS appears constant (Fig. 2). The interaction term between recanalization and serum BG was significantly assessed by a multivariable linear regression model with absolute NIHSS changes as a dependent variable suggesting that serum BG might modify the treatment effect of recanalization on short-term functional outcome. The reasons for this could be increased edema formation, a higher risk of bleeding complications, and increased oxidative stress and inflammation. In comparison, there was no significant treatment effect modification of recanalization on ENI and NIHSS changes in patients distinguished by long-term glucose profiles (low versus high HbA1c levels). In summary, these findings suggest that acute hyperglycemia may be directly linked to the stunned brain phenomenon in ischemic stroke. Considering the high number of patients with known or unknown [12] diabetes or prediabetes in the aging population, this phenomenon is relevant for estimating treatment effects and for early clinical prognosis.

In the past, it has been observed that a relevant proportion of ischemic stroke patients show delayed neurological recovery after stroke even after successful reperfusion. The reasons given were kidney disease, general anesthesia, or longer treatment intervals [9]. In ischemic stroke, serum BG is usually tested directly on admission, while HbA1c levels are not always routinely tested, or tested later in the clinical course. Hence, these parameters currently do not have any impact on clinical treatment decision-making. Diabetes is a major risk factor for stroke and is associated with significantly worse outcomes. [13] It is also known that approximately 23–53% of stroke patients have prediabetes, while the proportion of patients with diabetes is 14–46%. This is expected to result in a higher stroke burden in the future [14, 15].

Second, the formation of early ischemic edema has been suggested as a link between higher BG and worse outcomes, mainly due to alterations of the blood–brain barrier permeability. [16] Furthermore, high blood glucose levels also increase oxidative stress and cause inflammation, which can significantly reduce the efficacy of reperfusion treatment and, without rapid reperfusion, can cause significantly more tissue injury [17, 18]. Increased oxidative stress can lead to an increase in reactive oxygen species and other proinflammatory cytokines, which may also be associated with increased cerebral edema [19, 20]. In the later course, the immune system is known to be activated and cytokines and other inflammatory mediators are released. Higher BG could exacerbate this inflammatory response and thus contribute to a poorer functional outcome [21].

Altered autoregulatory processes may constitute another reason for a delayed neurological response after reperfusion in patients with elevated BG [22]. A study on intensive blood pressure lowering in patients with type 2 diabetes showed that microvascular complications lead to impaired autoregulation during blood pressure elevation, whereas patients without diabetes were able to maintain autoregulation by adjusting their blood pressure [24, 25]. Prior studies also illustrated that patients with established diabetes exhibited an imbalance in the coagulation and fibrinolysis system, resulting in a prothrombotic state characterized by increased platelet sensitivity, coagulation abnormalities, and hypofibrinolysis [12]. As a result, denser fibrin networks are more difficult to dissolve and antifibrinolytic proteins accumulate in the clot [23]. This association may have a direct impact on the response to endovascular treatment and delayed recovery, but it could also provide a potential approach for adjuvant therapy.

To our knowledge, this was the first study to investigate the effect of short- and long-term blood glucose levels on the treatment effect of endovascular recanalization on ENI, NIHSS changes, and final functional outcome. Despite confirming the beneficial effect of recanalization on short- and long-term outcome we observed that high BG was significantly associated with failure of ENI and NIHSS changes despite similar odds of reaching functional independence at day 90 and hence, suggesting a direct link to the “stunned brain phenomenon”. This phenomenon may be an explanation for delayed recovery of brain function due to a variety of mechanisms [24]. Future research is necessary to identify other possible variables that have an influence on this phenomenon in ischemic stroke patients undergoing endovascular treatment.

Currently, investigating adjuvant and pharmacological treatment options is a major focus in stroke research. In particular, potentially modifiable parameters that influence functional outcome and success of reperfusion treatment are being examined [25]. Previously, the SHINE trial assessed the effect of intravenous glyburide, an antidiabetic drug, on functional outcome in ischemic stroke patients; however, only 13% of the included patients underwent endovascular therapy, which might directly limit the potential effect of the adjuvant treatment [26]. Further studies analyzed the effect of glyburide on lesion pathophysiology and observed that the application of glyburide was associated with a reduction in tissue water uptake and mass effect after a larger hemispheric infarct [27]. Currently, the CHARM trial showed that patients who received intravenous glibenclamide showed a lower likelihood of a poor functional outcome at 3 months, when compared to placebo. According to subgroup analysis, a better outcome was observed in patients with medium-large stroke volumes (80–130 mL), patients who received mechanical thrombectomy or IVT, and patients with a wake-up stroke. Still, patients undergoing MT required a further MRI before studying inclusion (i.e., drug administration), which is an important limitation of this study.

The limitations of our study include the relatively small number of patients due to the strict inclusion and exclusion criteria. Additionally, our study was retrospective, necessitating subsequent prospective confirmation. Furthermore, poststroke hyperglycemia is a fluctuating phenomenon, and a solitary blood glucose reading might not adequately encapsulate the intricacies of ischemic brain conditions.

## Conclusion

Serum BG was significantly associated with ENI, despite a similar effect of recanalization on the final functional outcome in patients with low versus high BG. Hence, serum BG might be an important indicator of the “stunned brain phenomenon”.

## Data Availability

The data that support the findings of this study are available from the corresponding author upon reasonable request.
